# Proof-of-Concept of a Millimeter-Wave Integrated Heterogeneous Network for 5G Cellular

**DOI:** 10.3390/s16091362

**Published:** 2016-08-25

**Authors:** Shozo Okasaka, Richard J. Weiler, Wilhelm Keusgen, Andrey Pudeyev, Alexander Maltsev, Ingolf Karls, Kei Sakaguchi

**Affiliations:** 1 AVC Networks Company, Panasonic Corporation, Yokohama 224-8539, Japan; 2 Department of Wireless Communications and Networks, Fraunhofer Heinrich Hertz Institute, Berlin 10587, Germany; richard.weiler@hhi.fraunhofer.de (R.J.W.); wilhelm.keusgen@hhi.fraunhofer.de (W.K.); kei.sakaguchi@hhi.fraunhofer.de (K.S.); 3 Intel Corporation, Turgeneva Street, 30, Nizhny Novgorod 603024, Russia; andrey.pudeyev@intel.com (A.P.); alexander.maltsev@intel.com (A.M.); 4 Department of Bionics and Statistical Radiophysics, Lobachevsky State University of Nizhny Novgorod, Nizhny Novgorod 603950, Russia; 5 Intel Deutschland GmbH, Am Campeon 10–12, Neubiberg 85579, Germany; ingolf.karls@intel.com; 6 Department of Electrical and Electronic Engineering, Tokyo Institute of Technology, Tokyo 152-8552, Japan

**Keywords:** 5G cellular networks, millimeter wave, heterogeneous networks, flexible backhaul

## Abstract

The fifth-generation mobile networks (5G) will not only enhance mobile broadband services, but also enable connectivity for a massive number of Internet-of-Things devices, such as wireless sensors, meters or actuators. Thus, 5G is expected to achieve a 1000-fold or more increase in capacity over 4G. The use of the millimeter-wave (mmWave) spectrum is a key enabler to allowing 5G to achieve such enhancement in capacity. To fully utilize the mmWave spectrum, 5G is expected to adopt a heterogeneous network (HetNet) architecture, wherein mmWave small cells are overlaid onto a conventional macro-cellular network. In the mmWave-integrated HetNet, splitting of the control plane (CP) and user plane (UP) will allow continuous connectivity and increase the capacity of the mmWave small cells. mmWave communication can be used not only for access linking, but also for wireless backhaul linking, which will facilitate the installation of mmWave small cells. In this study, a proof-of-concept (PoC) was conducted to demonstrate the practicality of a prototype mmWave-integrated HetNet, using mmWave technologies for both backhaul and access.

## 1. Introduction

The fifth-generation mobile networks (5G) are expected to be deployed by 2020 and to be a primary source of Internet connection for the year 2020 and beyond. Driven by the rapid growth in the number of mobile-connected devices and the emergence of new services, research and development on 5G have been expanding [1,2]. The Working Party 5D (WP5D) of the International Telecommunication Union Radiocommunication sector (ITU-R) has established a work plan for International Mobile Telecommunications (IMT) for 2020 and beyond (IMT-2020) [3]. The 3rd Generation Partnership Project (3GPP) has initiated studies that aim to make functional specifications for 5G available in 2019 [4]. The leading 5G use case is enhanced mobile broadband, closely followed by critical and massive machine type communications used for wireless sensors, meters or actuators [5]. It is anticipated that 45 trillion networked sensors will be deployed in 20 years [6]. 5G should consider the ability to support a massive number of connected devices and various types of traffic.

The requirements for 5G include extremely high data rates, very low latency and the capacity to handle numerous devices, ultrahigh reliability, energy efficiency and reasonable costs [1]. Dealing with ever-growing traffic demand is one of the most important requirements. A 1000-fold increase in traffic is expected between 2010 and 2020 or 2025, with a further 10–100-times growth in the period from 2020 to 2030 [7,8]. To fulfill all of the requirements at reasonable cost, 5G is expected to require a heterogeneous network (HetNet) architecture, comprising multiple radio access technologies (RATs). The 5G HetNet will involve both improvement of existing RATs, such as 3GPP Long Term Evolution (LTE)-Advanced Pro and non-3GPP radios, like Wi-Fi, as well as the development of novel RAT(s) specifically designed to meet the 5G requirements. The HetNet architecture offers a promising approach by using a range of RATs in a flexible manner.

To achieve a greater than 1000-fold capacity improvement, the following three primary approaches [9] will need to be combined: (1) improvements in spectrum efficiency by advanced modulation or antenna techniques, such as massive multiple-input multiple-output (MIMO), non-orthogonal multiple access (NOMA) and multiuser superposition transmission (MUST) [9,10,11]; (2) expansion of the bandwidth by using high-frequency bands, including both centimeter- and millimeter-wave (mmWave) bands [12]; and (3) an increase in spectrum reuse by network densification [13], for example by deploying many small cells overlaid on a macrocell.

In this contribution, we focused on mmWave-integrated HetNet, in which novel mmWave technologies are introduced into traditional cellular networks [14,15]. Unlike the traditional cellular frequencies below 6 GHz, mmWave signals are inappropriate for long-distance transmission because they suffer from higher propagation losses and blockages. However, the mmWave bands offer more than 1 GHz of contiguous spectrum and are capable of delivering multi-gigabit per second (Gbps) data rates. Solutions combining massive MIMO with beamforming, in which a large number of antennas compensate for the propagation losses, are becoming increasingly feasible as antenna sizes become smaller and chip-scale antenna solutions become available [14,16]. These make the mmWave attractive for use on small cells in dense deployments.

A key challenge in the deployment of a large number of small cells is the provisioning of a backhaul connection [17]. Backhaul is a transport link of aggregate communication signals/data from macrocell or small cell base stations to the core network. Optical fiber links might be too expensive or even impossible, in view of the large number of locations. An alternative is the use of wireless backhaul. The high data rates of the access links incur equally high requirements on the backhaul, in terms of both the data rates and latency. MmWave communications with high gain antenna technologies offer flexible wireless backhaul with suitably high data rates [18,19,20,21].

This contribution presents our pioneering work on a proof-of-concept (PoC) of mmWave-integrated HetNet. The research and PoC were conducted within a joint European-Japanese research project named Millimetre-wave Evolution for Backhaul and Access (MiWEBA) [22]. The MiWEBA project focuses on the effective use of mmWave technologies in HetNet and especially on 60-GHz technologies. The envisioned HetNet comprises a conventional macro-cellular network and novel small cells that use the mmWave both for backhaul and access. In addition, we introduced into the HetNet the concept of control plane (CP) and user plane (UP) splitting [14,23]. In a CP/UP splitting HetNet, the CP is provided by a conventional macrocell to deliver broad coverage, while the UP data are provided by mmWave small cells. This architecture enables continuous connectivity with capacity boosting by using the advantages of both the macrocell and the small cells.

Some PoCs have already been conducted on mmWave technologies for 5G cellular networks. Early 2000 saw the first development of 60-GHz access by Communications Research Laboratory (CRL, now National institute of Information and Communications Technology, Tokyo, Japan) [24], achieving the world’s fastest data rate of 128 Mbps at that time. The main limit on the data rate was the processing speed at the baseband. The CRL study [24] encouraged the creation of the standard IEEE 802.15.3c in 2009, the first 1-Gbps over wireless communication standard [25]. This standard defined the current frequency regulation of the 60-GHz band. However, the actual use of the 60-GHz band only became a reality a few years later with the development of a complementary metal oxide semiconductor (CMOS) radio frequency integrated circuit (RFIC) with low-phase noise [26], built-in self-calibration [27] and an RF phase shifter with low power consumption [28]. The latest wireless standard for the 60-GHz band is IEEE 802.11ad (also referred to as WiGig), which was standardized in 2012 [29,30]. Intel (Santa Clara, CA, USA) then released the first WiGig chipset supporting 7 Gbps for docking stations, and in 2014, Qualcomm (San Diego, CA, USA) released a processor combining the LTE and WiGig standards [31,32]. The first WLAN AP product with the WiGig standard is scheduled for release in 2016 [33]. Samsung (Suwon, South Korea) and Deutsche Telecom (Bonn, Germany) have recently demonstrated 5G on a smartphone integrating 60-GHz technologies [34].

Although the regulations for mobile use have not yet been defined, research and development on mmWave frequencies other than a 60-GHz band is currently being undertaken. Samsung and Qualcomm have investigated the 28-GHz band [34,35,36], and Huawei (Shenzhen, China) and Nokia (Uusimaa, Finland) have been working on the 73-GHz band [37,38], mainly for 5G outdoor access. Two United States cellular operators, Verizon (Basking Ridge, NJ, USA) and T-Mobile (Bellevue, WA, USA), have applied for test radio licenses for the 28- and 38-GHz bands [39], and Ericsson (Stockholm, Sweden), Intel, Samsung, Qualcomm and Nokia are planning to join these tests. As well as mmWave access, much work is being conducted on mmWave backhaul technologies for 5G. InterDigital (Wilmington, DE, USA) has investigated 60-GHz meshed backhaul for small cell BSs in which a self-organizing function is realized by a phased array [40]. NEC (Tokyo, Japan) has released an E-band (71–86 GHz) transceiver supporting 10-Gbps backhaul to enable centralized radio access networks (C-RANs) [41]. This range of activity demonstrates the key role of mmWave technology in boosting the data rates of 5G systems. However, to date, no integrated PoC has been developed for 5G, which combines mmWave access, mmWave backhaul and mmWave antennas. To the best of the authors’ knowledge, this contribution is the first to demonstrate a PoC for an mmWave-integrated cellular network.

The rest of this paper is organized as follows. Section 2 introduces the system architecture and the components of the mmWave-integrated HetNet. Section 3 discusses state-of-the-art mmWave antenna technologies for backhaul and access. Section 4 presents our PoC of the mmWave-integrated HetNet. Finally, Section 5 presents our conclusions.

## 2. mmWave-Integrated Cellular Networks

### 2.1. Control/User Plane Splitting for HetNet

In CP/UP splitting [14,23,42], also referred to as “phantom cell” or “soft cell”, the CP and UP are handled separately. In a CP/UP-split HetNet, the mobile stations (MS), also referred to as user equipment (UE), are capable of maintaining a logical link with both the macrocell and multiple small cells. This capability, called dual connectivity (DC), allows the CP and UP to be handled individually and flexibly. The single CP also enables efficient, centralized radio resource control (RRC), for example mobility control or beamforming (BF). DC affects UE peak power consumption because the UE has to activate multiple RF devices at the same time. However, the higher throughput makes the download time shorter, allowing the device to go to the sleep state soon after the download has been finished. In [43,44], it is indicated that average UE power consumption with two frequency carrier aggregation does not exceed that with single frequency carrier. Furthermore, a 99.7% reduction of UE power consumption could be achieved via a combination of traffic offloading and dynamic on/off [45].

**Figure 1:**
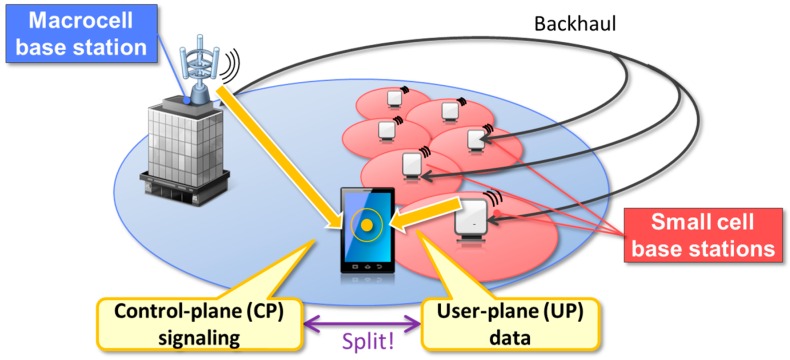
CP/UP splitting in the C-RAN-based HetNet architecture.

To achieve CP/UP splitting, the macrocell and small cells are connected via a backhaul interface used for exchanging control information and user data and are controlled by the same RRC entity as shown in Figure 1. The backhaul, as the intermediate link between the cells and the centralized network, is therefore an essential part of the CP/UP-split HetNet. The use of mmWave technologies in the backhaul is discussed in Section 3.

The DC and CP/UP splitting feature was introduced in Release 12 of the 3GPP LTE standard [42]. In Release 13, it was extended to support DC between the LTE and an IEEE 802.11 wireless local area network (WLAN) [46,47]. The detailed architecture of LTE-WLAN CP/UP splitting in Release 13 is discussed in Section 2.3. The CP/UP splitting concept must be a key enabler of HetNet.

### 2.2. 60-GHz mmWave WLAN

At the World Radiocommunication Conference 2015 (WRC-15), ITU-R identified portion(s) of the frequency range between 24.25 and 86 GHz to be considered for allocation to IMT-2020 [48]. However, the 3GPP LTE standards before Release 13 were designed for frequency bands below 6 GHz [49] and are not optimized for the mmWave frequency bands. In contrast, IEEE 802.11ad WLAN [29], also known as WiGig (wireless-gigabit) [30], addressed the unlicensed 60-GHz frequency band, which has at least 5 GHz of continuous bandwidth available in many countries [50]. WiGig uses 2.16 GHz-wide continuous bands per channel for transmission and is capable of multi-Gbps transmission. WiGig supports three distinct modulation methods: spread-spectrum, single-carrier (SC) and orthogonal frequency-division multiplex (OFDM) modulations. The spread-spectrum modulation is dedicated to the transmission of control messages. SC modulation is mandatory and is suitable for low-power consumption devices because of the low peak-to-average power ratio (PAPR). OFDM modulation is optional, suitable for relaxed power-consumption devices and more robust against multipaths than SC modulation. An example of the modulation and coding schemes (MCS) for WiGig is shown in Table 1. The physical (PHY) layer achieves a maximum data rate of almost 7 Gbps, and the next generation IEEE 802.11ay 60-GHz WLAN [51] aims to support a maximum throughput of at least 20 Gbps on the medium access control (MAC) layer by spatial multiplexing or further improvement of MAC efficiency.

**Figure 2:**
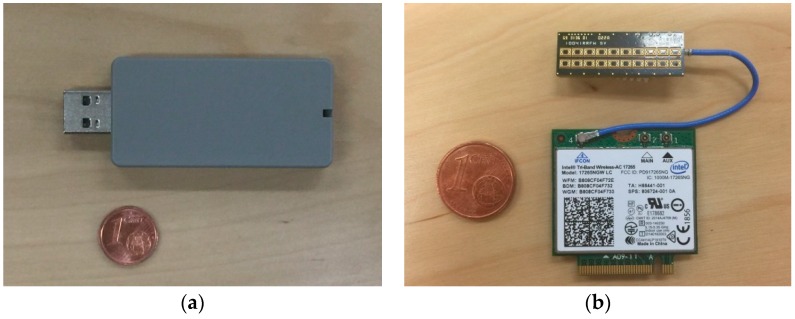
IEEE 802.11ad/WiGig communication devices: (**a**) Panasonic^®^ WiGig-USB dongle: 60-GHz antennas, RF, PHY and MAC components are bundled into one package. Universal Serial Bus (USB)-3.0 is used as the interface to higher layer processors, such as a personal computer (PC). (**b**) Intel^®^ Wireless Gigabit Antenna module (top) and Intel^®^ Tri-Band Wireless-AC 17265 WiGig and Wi-Fi + Bluetooth combination module [52] (bottom). The antenna module contains a beamforming phased antenna array (PAA) of 10 × 2 elements (8 × 2 active elements, 8 in azimuth and 2 in elevation).

The latest semiconductor technology brings 60-GHz communication to small handsets. For example, silicon-based CMOS technologies are implementing integrated system-in-package systems including mixers, LNAs and PAs in the mmWave frequency bands [14]. Figure 2 shows examples of IEEE 802.11ad/WiGig-compliant communication devices.

### 2.3. LTE-WLAN Interworking and Aggregation

The access network discovery and selection function (ANDSF) [53,54,55,56] is a core-network (CN) function that controls UP data offloading between 3GPP RATs (e.g., LTE) and non-3GPP RATs (e.g., WLAN). ANDSF is located in the evolved packet core (EPC), which is part of the 3GPP CN. ANDSF delivers to the UEs the auxiliary information for RAT discovery and selection on the basis of UE context, such as location or IP flow. On the basis of the delivered policies, the UE selects an appropriate RAT to connect with the network.

**Figure 3:**
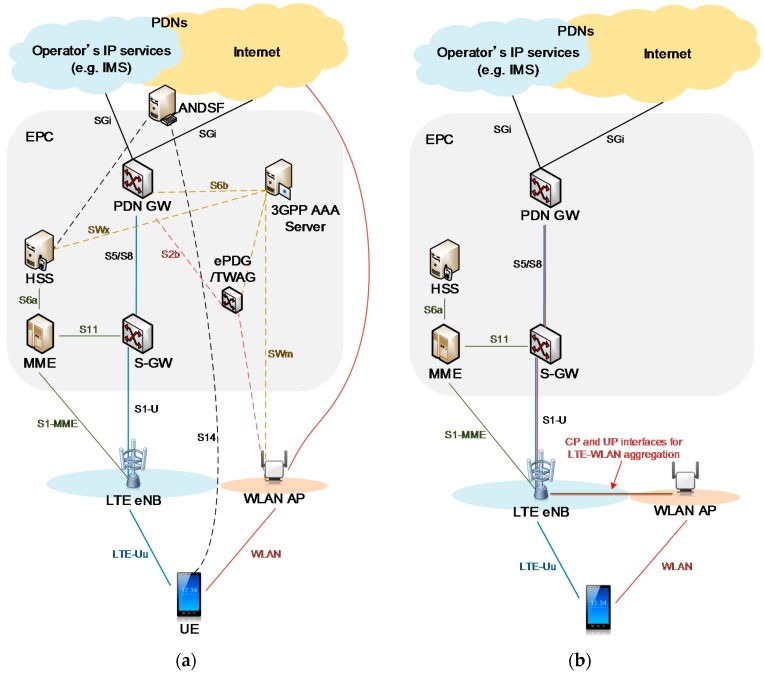
Example LTE-WLAN coordination and integration architectures: (**a**) 3GPP/WLAN interworking architecture and (**b**) LTE-WLAN aggregation.

Figure 3a shows an example of the ANDSF-based LTE-WLAN interworking architecture known as CN-level interworking. In this LTE network, the UE is connected to a base station (BS) called evolved Node B (eNB), via an LTE air interface, which is called a Uu interface (radio interface between the Universal Terrestrial Radio Access Network and the UE). When the traffic from an external packet data network (PDN), for example the Internet, is handled by the Uu interface, the traffic is transferred via the PDN gateway (PDN GW), the serving gateway (S-GW) and eNB to the UE. For offloading the traffic to WLAN, two schemes are available:

- Non-seamless WLAN offload (NSWO): In NSWO, the traffic is not sent via the EPC, but directly from the Internet to the WLAN-AP. An offloading scheme that bypasses the EPC is called non-seamless WLAN offload (NSWO). In NSWO, the IP flow between the network and UE is interrupted during LTE-WLAN handover, because a different IP address will be allocated to each RAT. In addition, the UE cannot use the operator’s private IP services via WLAN.
- Seamless WLAN offload: When the UE is capable of IP flow mobility (IFOM), the traffic can be transferred to the UE via the WLAN-AP and a PDN GW, an evolved packet data gateway (ePDG) or a trusted wireless access gateway (TWAG). In this scheme, the UE can offload the same IP flow from LTE to WLAN. The PDN GW is an anchor point between the LTE and WLAN accesses.

ANDSF is an efficient and scalable mechanism for controlling the UE behavior of RAT discovery and selection from the network. The UE can be authenticated from both LTE and WLAN networks with a single subscriber identity because the EPC and WLAN can exchange authentication, authorization and accounting (AAA) information via a 3GPP AAA server. However, ANDSF cannot control UP offloading in a hyper-dense HetNet for two reasons. First, the initial design of Release 8 ANDSF was intended to provide policies based on a static coverage map [57] and not to be responsive to real-time traffic or radio network conditions. Later releases of ANDSF were extended to provide quasi-real-time statistics, such as RAN congestion. However, it is difficult to handle real-time changes in a dense network because the ANDSF entity is separated from both eNB and WLAN AP in the network. Second, the mobility anchor point (PDN GW) is too distant from the eNB and WLAN AP in the network. Frequent inter-RAT handover in a dense network consequently increases the signaling load in the EPC, which lengthens the handover interruption time. Moreover, the enhanced features deployed in eNB, such as mobile-edge computing (MEC) [58], cannot be used via WLAN access.

An RAN-level LTE-WLAN integration architecture [46,47,59] is therefore proposed and is shown in Figure 3b. In this architecture, the LTE eNB and WLAN AP are directly connected for both UP and CP via a coordination interface that directly transfers real-time traffic and radio information. The mobility anchor point resides in the eNB, minimizing interruptions during handover. This architecture enables CP/UP splitting between the LTE and WLAN since a single CP entity in eNB (i.e., RRC) can handle both LTE and WLAN UPs.

**Figure 4:**
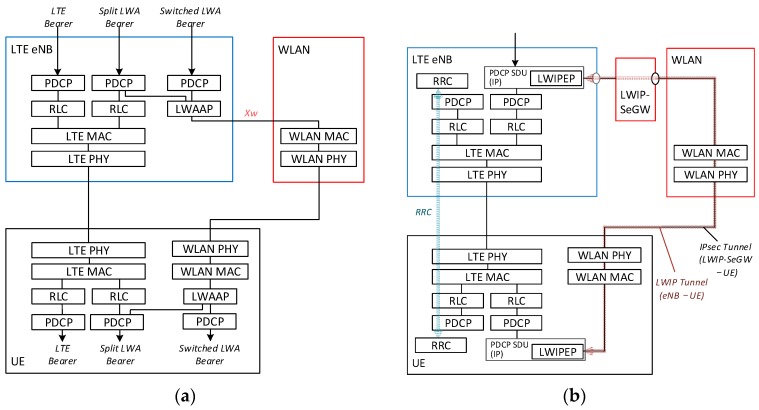
LTE-WLAN interworking architectures in Release 13: (**a**) LTE-WLAN aggregation (LWA) in a non-collocated scenario and (**b**) LTE-WLAN radio level integration with IPsec tunnel (LWIP).

The LTE-WLAN integration architecture was proposed and discussed during 3GPP standardization. As a result, two types of LTE-WLAN integration architectures [46,47] are defined in Release 13: LTE-WLAN aggregation (LWA) and LTE-WLAN radio level integration with IPsec tunnel (LWIP). Figure 4 shows the protocol stacks of these architectures.

The main difference between the architectures is the layer of the forwarded packets. In LWA, the eNB forwards packet data convergence protocol (PDCP) data units (PDUs) to the WLAN AP. The PDCP PDUs, which are encapsulated in the LWA adaptation protocol (LWAAP), can be sent via WLAN. In this architecture, the PDCP features can be utilized in WLAN access. For example, security protection on WLAN can be simplified because the PDCP provides external ciphering and an integrity protection mechanism. However, the WLAN infrastructure needs to be updated to support WLAAP. In LWIP, the eNB forwards PDCP service data units (SDUs), i.e., IP packets, to the WLAN AP. The forwarded IP packets are encapsulated in the Generic Routing Encapsulation (GRE) protocol and are secured using IP security (IPsec). The setting of an LWIP bearer is therefore more complicated than that of LWA. However, LWIP can be introduced into the existing WLAN infrastructure because the GRE and IPsec tunnels are transparent to the existing infrastructure.

Both LWA and LWIP are controlled by eNB based on UE measurement reporting of radio information, so that their handover failure can be reduced dramatically as reported in [59].

## 3. mmWave Beamforming Antenna and Backhaul

Highly directional antennas are required to compensate the millimeter-wave propagation, which has a higher path loss than lower band communication systems, like LTE or Wi-Fi. These highly directional antennas should also be steerable to support access and a reconfigurable backhaul link. A range of concepts and designs for such antennas have been proposed [18,19,20,21]. In the next two subsections, we discuss the most practical approaches.

Different requirements are placed on the mmWave antennas in backhaul and access. For small cell backhauling, medium distances of several hundred meters in static configurations are most important, while access links require fast beamsteering to track mobile users.

The key performance indicators for backhaul links are the data rate, power consumption and cost. The cost can be divided into capital expenditure (CAPEX) and operational expenditure (OPEX). While CAPEX is driven by the price of the device and the cost of installation, OPEX is driven by power consumption, licensing costs and required servicing.

### 3.1. Passive Reflectarray Antennas

Our implementation of a static mmWave backhaul link was based on the use of passive reflecting structures [20]. These so-called reflectarrays comprise an array of specially-designed dipole elements on a printed circuit board (PCB), with a ground metallization layer on the opposite side [60]. Through the proper design of the dipole structures, the phase of the locally-reflected electromagnetic wave can be influenced, and a collimating effect similar to that of a parabolic dish can be obtained. The dipole elements can be made polarization dependent, allowing the reflectarray to use different focal points for the orthogonal polarizations. Furthermore, the shape of the collimated beam can be controlled by the phase configuration. The aperture and gain of a reflectarray can be adapted to match specific needs, in the same way as a parabolic dish. The application of well-understood PCB technology allows both the cost and weight to be kept low.

**Figure 5:**
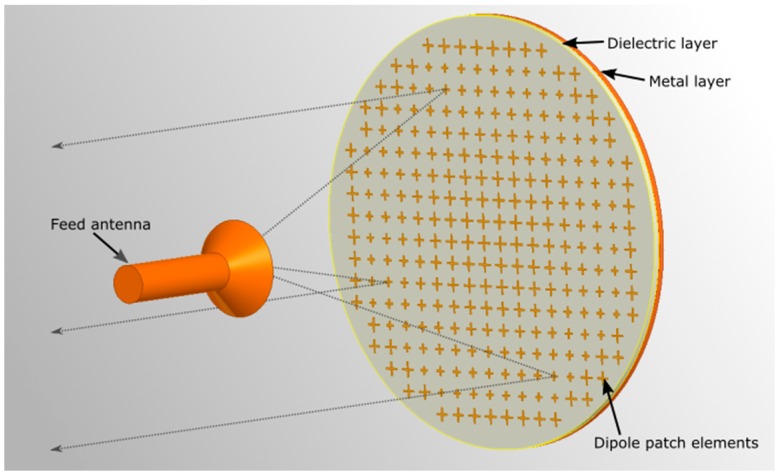
Static backhaul solution based on reflectarray technology.

We combined an integrated mmWave transceiver with on-chip antennas in a reflectarray structure, with the goal of increasing the antenna gain and therefore the link distance that could be achieved. Figure 5 shows our design. The integrated transceiver chip was installed at the position of the feed antenna and facing towards the array.

The integrated transceiver can be, for example, based on the IEEE 802.11ad standard, operating at 60 GHz. RF feeding structures and their associated losses can be minimized, as the on-chip antenna requires only a low gain. As the proposed solution is intended for static links, a mechanical fine alignment can be implemented to reduce the installation cost.

**Figure 6:**
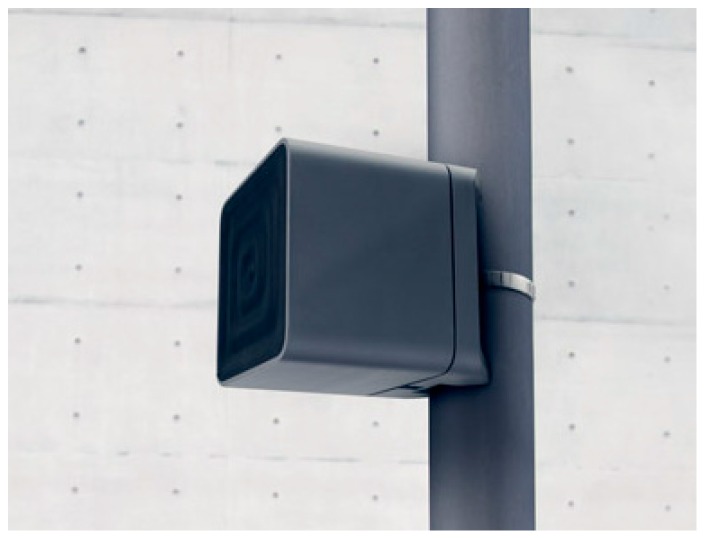
Prototype of the backhaul link installed on a lamp post.

A prototype of the static backhaul is shown in Figure 6. The transceiver was based on an IEEE 802.11ad modem and was connected to an embedded PC acting as both the controller and router. A standard Gigabit Ethernet connection was available. Average and peak power consumption of the prototype was 14 Watts and 20 Watts, respectively. Since the IEEE 802.11at power over Ethernet (PoE) standard allows up to 25.5 Watts (0.6 A × 42.5 V) of power delivered at power devices [54], the entire device could be supplied with PoE.

The achievable net TCP/IP throughput of the point-to-point prototype was around 1.7 Gbps, and the round trip IP latency was below 1 ms. The achievable range was in the order of several hundred meters using a reflector with a 20-cm edge length. The data rate could be partitioned arbitrarily between uplink and downlink, as the underlying protocol operated in TDD mode. However, the wired Ethernet interface limited the speed in each direction to around 1 Gbps. Full throughput was therefore achieved when traffic was symmetrical in both directions.

### 3.2. Highly Directional Steerable mmWave Antennas

**Figure 7:**
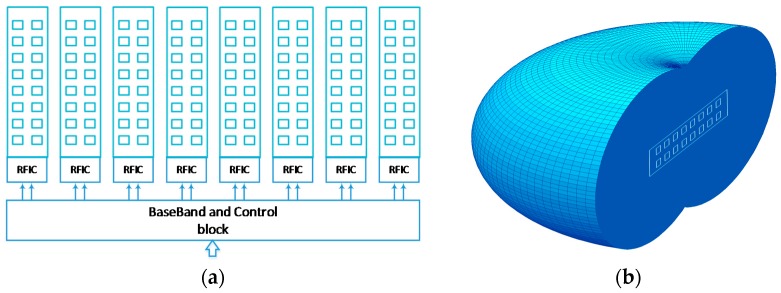
Highly-directional steerable mmWave antennas: (**a**) modular antenna array architecture and (**b**) lens array antenna scheme.

Phased antenna arrays are a well-established and proven technology for electronically-steerable antennas. However, highly directional arrays with large apertures may encounter a range of implementation problems, such as heat dissipation, feeding line losses and placement/installation difficulties. These are especially critical for small-form factor millimeter wave antennas. In [21], two novel solutions were proposed: the modular antenna array (MAA) architecture and the lens array antenna (LAA). The first allows the creation of a large aperture, high gain antenna array from small-phased antenna array (PAA) modules (see Figure 2b). Hybrid beamforming [61] can be realized in the MAA [62] through coarse analog phase-shifting in the RFIC circuits and fine digital beamforming in the baseband (BB) (see Figure 7a). In this design, an arbitrary number of single modules (eight in this case) is connected to a single baseband and control block.

This proposed design may substantially decrease the feeding line power losses, as they are of a short length in each subarray, and the common feeding is performed at an intermediate frequency (IF). Mass production of unified inexpensive modules further reduces the cost of the large-aperture antenna. The total output power is equal to the sum of the individual single modules and can therefore be scaled according to the specific needs.

The LAA architecture uses a different approach to achieve high directivity with a phased antenna array. The array aperture is increased by using a dielectric lens with a special geometry [63,64,65] that focuses the single module phased array beam into a single plane (the elevation, for example), allowing steering in another plane (the azimuth). For efficient azimuth plane beamsteering, a lens array antenna with an elliptic-toroidal geometry is proposed [21] with the emitting-phased antenna array module placed on the backside (Figure 7b). In this antenna design, the antenna aperture is determined by (a) the vertical dimension of the lens and (b) the horizontal dimension of the phased antenna array. The number of array elements in the vertical plane may be significantly lower than is required for a phased antenna array of the same gain. Effective heat dissipation may be achieved by placing a radiator on the back of the lens array antenna.

The LAA prototype developed in the framework of the MiWEBA project has an aperture (lens height) of 112 mm and a gain of up to 27 dBi. To demonstrate the feasibility of the proposed LAA in access and backhaul applications, real packet transmissions with different MCSs were performed under field conditions. Three experimental scenarios were investigated: device to device (D2D) linking (single PAA module to single PAA module transmission), access linking (LAA to single PAA module) and long-range backhaul (LAA to LAA).

The use of single modules without lenses (D2D) allowed transmissions with the highest data rate in SC mode (MCS #12: 4.7 Gbps physical layer rate, 16-QAM with coding rate ¾; see Table 1) at distances up to 15 m. The application of a lens at the transmitter side achieved the maximum data rate (4.7 Gbps), making 16 QAM modulation practical for outdoor communications at distances of about 30–35 m, which can be appropriate for millimeter wave access links.

**Figure 8:**
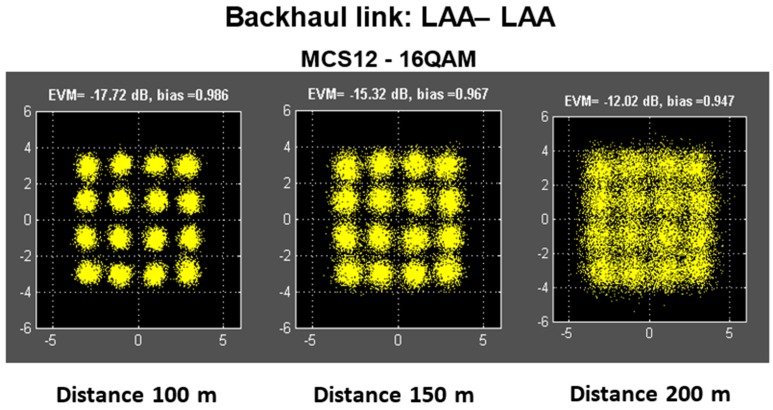
Constellation diagrams for the backhaul link with an LAA at the TX and RX side.

A second experiment investigated the backhaul link on the base of the two LAA antennas. Figure 8 gives a screenshot of the constellation diagrams obtained for MCS #12 transmissions at 100, 150 and 200 m. It was demonstrated that the backhaul link with two LAA gave the highest data transmission rate of 4.7 Gbps at distances up to 200 m, with a negligible packet error rate.

**Figure 9:**
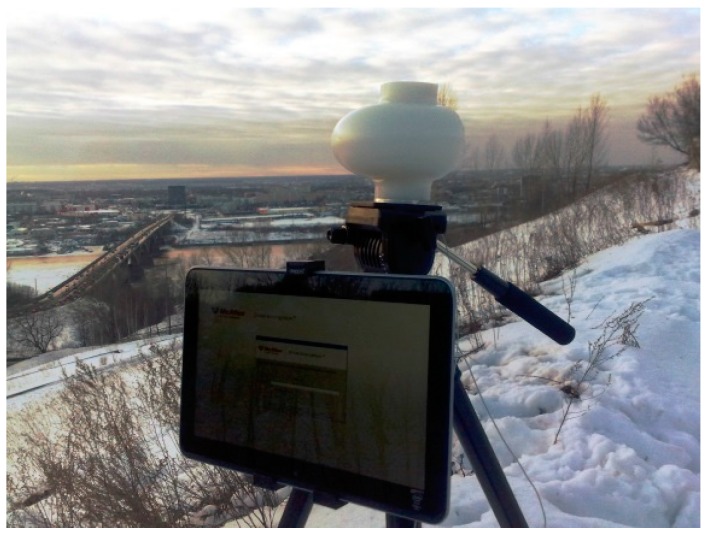
LAA prototype link in the field trial.

The outdoor field trial setup for LAA is shown in Figure 9. This study demonstrated that the LAA is a practical high-gain steerable millimeter wave antenna for use in future millimeter wave overlay networks (D2D, access and backhaul) within the framework of 5G communication systems.

In LAA, we have used one antenna array module with 2 × 8 elements including the RFIC chip, and one BB module for digital signal processing. The total power consumption was about 4–6 Watts (from direct measurements) depending on regime of work (RX or TX). For the MAA prototype, we have used eight antenna array modules (each with 2 × 8 elements and one RFIC chip) and one, common, BB module for digital signal processing. Thus, the total power consumption of the eight-module MAA was estimated as 20–25 Watts. Therefore, both the LAA and MAA equipment could be power supplied with PoE.

## 4. Proof of Concept Implementation and Integration

**Figure 10:**
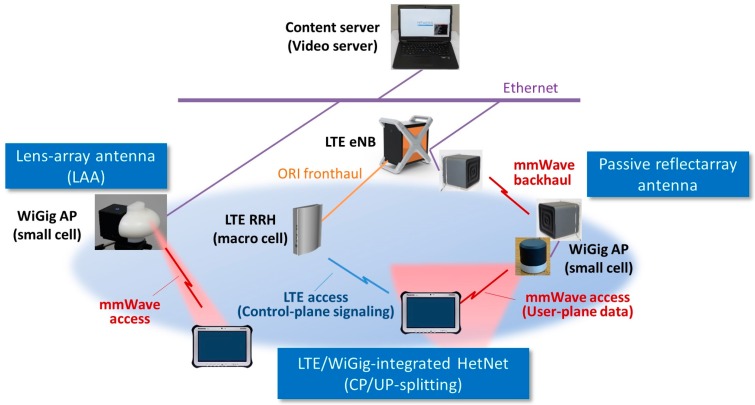
Overall illustration of mmWave-integrated HetNet PoC.

**Figure 11:**
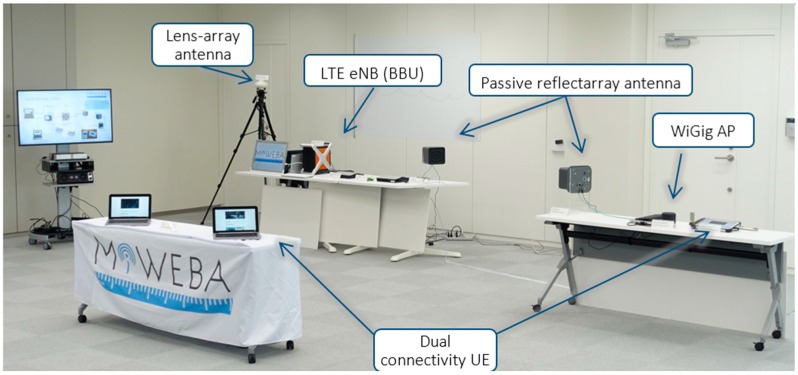
Hardware set-up of HetNet PoC.

An overview of our PoC is given in Figure 10 and Figure 11. The PoC hardware included the backhaul and access equipment. IP-based wireless backhaul linking was established using a reflectarray antenna with an integrated WiGig transceiver. LAA was used for access. A HetNet access network was used, with an access link provided in both 2-GHz LTE and 60-GHz WiGig. CP/UP splitting was introduced in the HetNet. A multi-RAT CP application implemented in LTE eNB managed UP data offloading between the LTE and WiGig.

### 4.1. LTE/WiGig-Integrated HetNet Prototype

**Figure 12:**
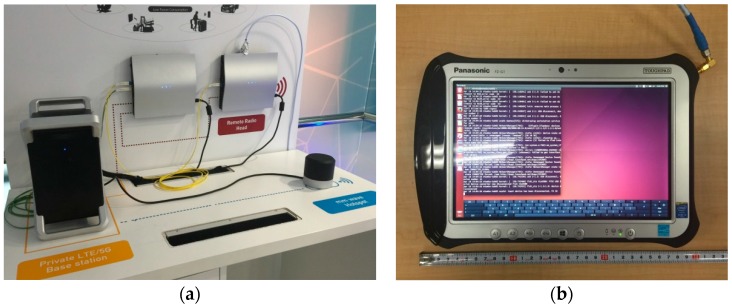
PoC for LTE-WiGig-integrated HetNet: (**a**) LTE eNB, RRH and WiGig AP and (**b**) dual-connectivity UE with LTE and WiGig modules.

Our PoC hardware for the mmWave-integrated HetNet used an LTE eNB as the macrocell and WiGig APs as the small cells. Figure 12 is a photograph of our PoC hardware. The LTE macrocell comprised an eNB baseband unit (BBU) and a remote radio head (RRH) connected via an optical fronthaul based on the European Telecommunications Standards Institute (ETSI) Open Radio Interface (ORI) standard [66,67]. This is capable of transferring LTE baseband I/Q signals with a 50% compressed data rate. The WiGig AP was connected to the LTE eNB via Ethernet. The UE had LTE modem and WiGig devices were implemented. The WiGig device is the shown in Figure 2 and supported MCSs up to Index #9, as shown in Table 1.

**Figure 13:**
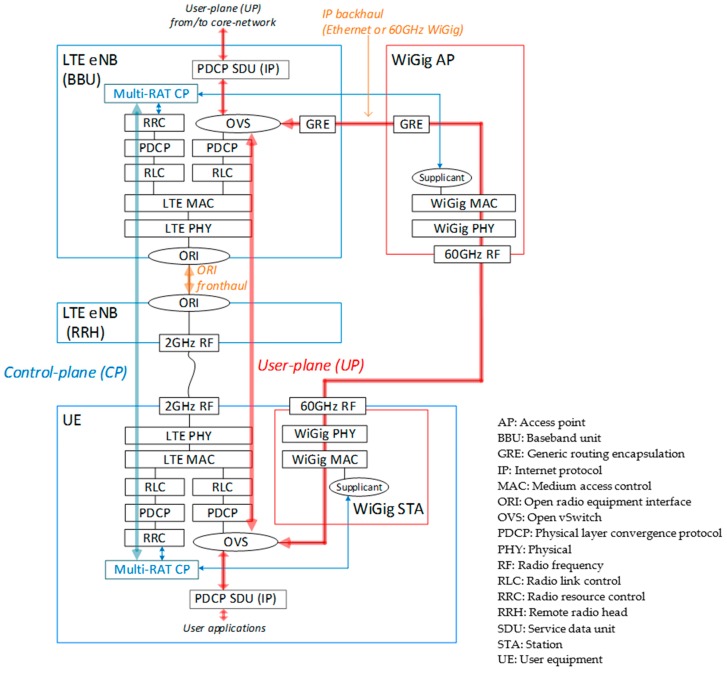
Protocol stack of LTE-WiGig-integrated HetNet PoC.

Figure 13 shows the protocol stack of the PoC. This was similar to LWIP, but with the security protections omitted for simplicity. Security is an important feature of mobile networks, but was not the focus of the current PoC. We will return to this feature in future work. An LTE-WiGig-integrated CP (multi-RAT CP) application was implemented on top of the CP protocol stack. The multi-RAT CP monitored the real-time traffic load and radio link conditions, such as the received signal-strength indicator (RSSI) of both the LTE and WiGig. The measured link condition on UE was reported to the eNB by CP signaling via LTE. On the basis of the monitored information, the multi-RAT CP on the eNB managed the handling of the UP data. The UP data path was switched between LTE and WiGig by Open vSwitch (OVS) [68]. When the WiGig was used, the multi-RAT CP controlled activation and authentication of the WiGig. PDCP SDUs were sent via the WiGig or LTE link. Since the interface between the LTE-eNB and WiGig-AP was IP-based, various Layer-2 media can be used for the backhaul, including the 1, 2.5, 5 or 10 Gigabit Ethernet or mmWave backhauls presented in Section 3.1 and Section 3.2.

**Figure 14:**
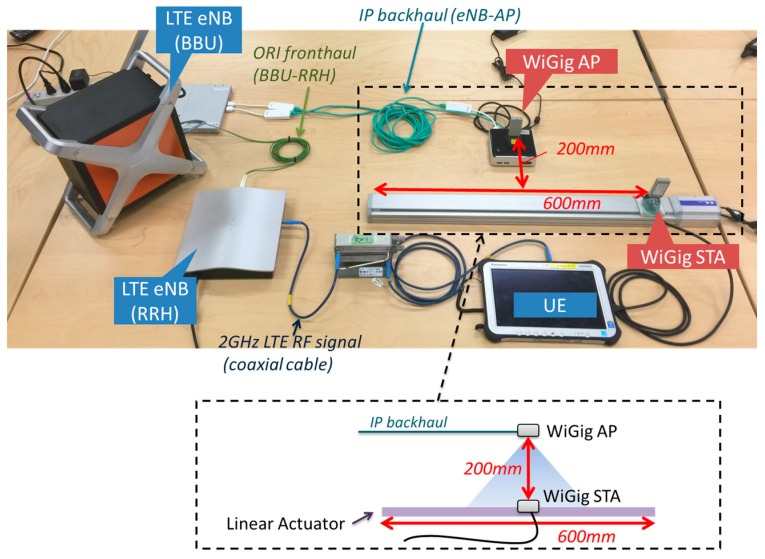
Experimental setup.

To demonstrate the CP/UP splitting concept, we evaluated the performance of LTE-WiGig handover. The experimental setup is shown in Figure 14. The WiGig station (STA) device was attached to a linear actuator and moved horizontally at a constant velocity of 83 mm/s. The vertical distance between the AP and STA antennas was set to 200 mm, so that the STA moved into and out of the coverage area of the WiGig. Switching of the UP data path was based on the availability of the WiGig link and the reported RSSI. If the WiGig link were available and the RSSI exceeded the threshold *RSSI_th_*, the UP data was offloaded to the WiGig link. Otherwise, it was carried on the LTE link.

**Figure 15:**
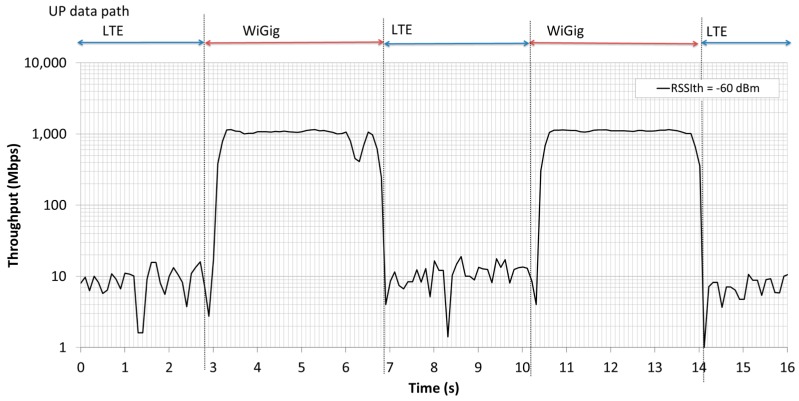
Transition of instantaneous UP throughput during LTE-WiGig handover.

Figure 15 shows the transition of the instantaneous UP throughput during an LTE-WiGig handover. The downlink UP data were transmitted from the eNB in TCP using Iperf [69]. The handover decision threshold *RSSI_th_* was set to −60 dBm, and the throughput was measured in the MAC layer. From the figure, it can be seen that the data path was switched smoothly, depending on the UE location, and that a throughput of more than 1 Gbps was achieved when the UP path was switched to WiGig.

### 4.2. PoC Applications

Two applications were selected to showcase the benefits of the mmWave-integrated cellular networks proposed by MiWEBA: “Context Aware Caching” and “Small Cell On/Off”.

**Figure 16:**
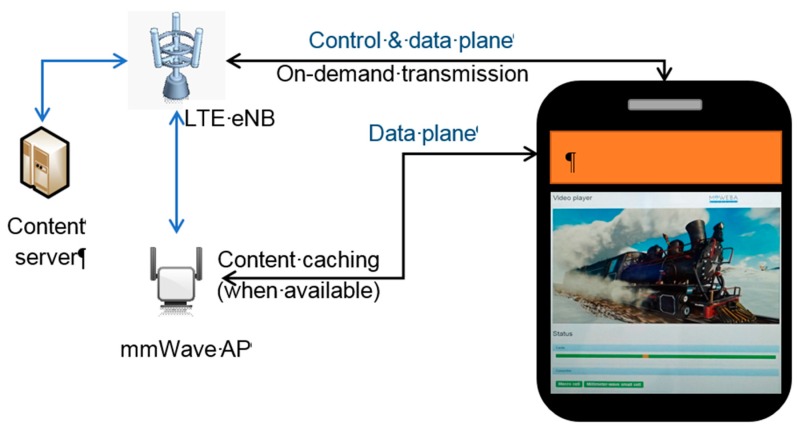
Caching PoC demonstration.

The setup for “Context Aware Caching” is shown in Figure 16. It comprises a dual-connectivity user device, a macro base station (LTE eNB) and an mmWave small cell (mmWave AP). The client runs a video streaming application while on the move. A context aware cache entity, lying as a middle layer between the video application and the network connection, performs the data requests, using the connectivity status (mmWave connection available or unavailable) as context information. The video file (served as multiple small chunks in Hypertext Transfer Protocol Live Streaming (HLS) format) is fetched from the content server and made available to the video player. When within the coverage of an mmWave small cell, the cache pre-fetches and stores the video file chunks locally at the maximum achievable rate. For this purpose, the cache creates a database of all chunks in the currently playing video. A status is associated with each chunk indicating whether it is locally available, currently downloading or not locally available. The most recently played chunk is stored as a pointer and used to decide which chunk is downloaded next. When outside the range of a small cell, the cache fetches the content on a just-in-time basis to reduce the load on the LTE link. From the user’s perspective, the content is available seamlessly. We demonstrated [70] “Context Aware Caching” by using the PoC and confirmed that the downloading rate was boosted dramatically while an mmWave link was available.

**Figure 17:**
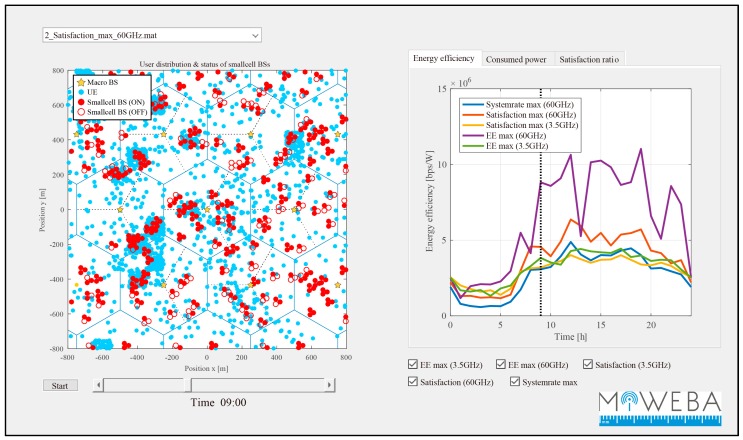
Snapshot of small cell on/off (**left**) and its energy efficiency (**right**).

The second demonstration of PoC was the ability to control and optimize the operation of the network resources in terms of energy efficiency by using the developed multi-RAT CP [71,72]. The centralized RAN (C-RAN), which is LTE eNB in this case, processes context information on its mmWave small cell base stations, including the number of connected user devices and their traffic demand. Depending on the load situation, the C-RAN turned off small cells via the multi-RA CP to save energy. As the user devices were always connected to the CP, the small cells could be immediately reactivated to meet traffic demand. Figure 17 shows a snapshot of small cell on/off and its energy efficiency. This is a result of a system level simulation of the mmWave-integrated cellular networks; however, the function used in this simulation can be realized by the PoC hardware shown in Section 4.1. The left of Figure 17 shows the status of small cell on/off, and the right shows the energy efficiency of the network measured by (bps/W). In the left, the distribution of UEs (blue circles) is not uniform, and there are several hotspots whose locations change in space and time. In this application, the C-RAN implemented in LTE macro eNBs (indicated by yellow stars in the figure) collects traffic demands from UEs. Based on this information, the C-RAN dynamically controls the status of small cell BSs as on (filled red circle) or off (unfilled red circle) to maximize the energy efficiency. In the case of on, the C-RAN immediately switches the UP to small cell BSs as described in Section 4.1. The right of Figure 17 shows the realized energy efficiency by changing time in a day. The performance is calculated with two different frequencies of 60 GHz and 3.5 GHz. Since the coverage (footprint) of 60 GHz is much smaller than 3.5 GHz, there is higher impact of small cell on/off in 60 GHz (purple line) than 3.5 GHz (green line). It is also clear that the performance of energy efficiency maximization (purple line) in the 60-GHz band has much better performance in terms of energy efficiency compared to that of system rate maximization (yellow line) in 60 GHz without the control of small cell on/off. In this demonstration, we just showed a snapshot of “Small Cell On/Off”; however, the details of optimization algorithm and simulation results can be seen in [71,72].

## 5. Conclusions

In this contribution, we disclosed a piloted proof-of-concept (PoC) of an mmWave-integrated heterogeneous network (HetNet) utilizing mmWave technologies both for backhaul and access for integration into cellular networks. In the HetNet, CP/UP splitting was implemented to allow user plane access in the mmWave small cells, while retaining a reliable control plane connection to the macro cell. Passive high-gain antenna technology in mmWave was applied for wireless backhauling of the small cells. A lens antenna array with steerable beam was used for mmWave access. This PoC was conducted to demonstrate the practicality of the mmWave-integrated HetNet. Successful CP/UP splitting between the conventional LTE and mmWave WiGig was demonstrated, and seamless connectivity was achieved between the different technologies. The high-throughput performance of the long-range backhaul link using a passive reflectarray was also confirmed. Although the mmWave access targets high-speed data communication in the first phase of 5G, the proposed architecture may be applied to future ultra-real-time and low latency sensor networks as required in autonomous driving that will be realized in the later phase of 5G. The achieved results will be contributed respectively to 3GPP, IEEE, ITU and ETSI.

## Figures and Tables

**Table:** 

Abbreviation	Term	Abbreviation	Term
3GPP	3rd generation partnership	IMS	IP multimedia subsystem
AAA	Authentication, authorization and accounting	IP	Internet protocol
ANDSF	Access network discovery and selection function	LTE	Long-Term Evolution
AP	Access point	MME	Mobility management entity
eNB	Evolved Node-B	PDN	Packet data network
EPC	Evolved packet core	S-GW	Serving gateway
ePDG	Evolved packet data gateway	TWAG	Trusted wireless access gateway
GW	Gateway	UE	User equipment
HSS	Home subscriber server	WLAN	Wireless local area network

**Table:** 

Abbreviation	Term	Abbreviation	Term
LWA	LTE-WLAN aggregation	MAC	Medium access control
LWAAP	LWA adaptation protocol	PDCP	Packet data convergence protocol
LWIP	LTE-WLAN radio level integration with IPsec tunnel	PHY	Physical
LWIPEP	LWIP endpoint	RLC	Radio link control
LWIP-SeGW	LWIP security gateway	SDU	Service data unit

**Table 1:** Modulation and coding scheme examples of IEEE 802.11ad (BPSK: Binary Phase-Shift Keying, QPSK: Quadrature Phase-Shift Keying, QAM: Quadrature Amplitude Modulation, SQPSK: Spread QPSK).

MCS Index	PHY Mode	Modulation	Coding Rate	PHY Data Rate (Mbps)
1	SC	π/2-BPSK	1/2	385
6	SC	π/2-QPSK	1/2	1540
9	SC	π/2-QPSK	13/16	2502.5
10	SC	π/2-16QAM	1/2	3080
12	SC	π/2-16QAM	5/8	4620
13	OFDM	SQPSK	1/2	693.00
15	OFDM	QPSK	1/2	1386.00
18	OFDM	16QAM	1/2	2772.00
22	OFDM	64QAM	5/8	5197.50
24	OFDM	64QAM	13/16	6756.75

## Abbreviations

3GPP	Third Generation Partnership Project
5G	Fifth generation mobile networks
AAA	Authentication, authorization and accounting
ANDSF	Access network discovery and selection function
AP	Access point
BBU	Baseband unit
BS	Base station
CAPEX	Capital expenditure
CP	Control plane
CPRI	Common public radio interface
CN	Core network
C-RAN	Centralized-RAN
D2D	Device-to-device
DC	Dual connectivity
eNB	Evolved Node B
EPC	Evolved packet core
ePDG	Evolved packet data gateway
GRE	Generic routing encapsulation
HetNet	Heterogeneous network
HSS	Home subscriber server
IEEE	Institute of Electrical and Electronics Engineers
IF	Intermediate frequency
IFOM	IP flow mobility
IP	Internet Protocol
IPsec	IP security
ITU-R	International Telecommunications Union-Radiocommunication Sector
IMS	IP multimedia subsystem
IMT-2020	International Mobile Telecommunications for 2020 and beyond
LAA	Lens array antenna
LNA	Low-noise amplifier
LTE(-A)	Long-Term Evolution (-Advanced)
MAC	Medium access control
MCS	Modulation and coding scheme
MEC	Mobile edge computing
MIMO	Multiple-input multiple-output
MME	Mobility management entity
MUST	Multi-user superposition transmission
NOMA	Non-orthogonal multiple access
NSWO	Non-seamless WLAN offload
mmWave	Millimeter wave
OFDM	Orthogonal frequency division multiplexing
OPEX	Operational expenditure
ORI	Open radio equipment interface
OVS	Open vSwitch
PAPR	Peak to average power ratio
PA	Power amplifier
PAA	Phased antenna array
PC	Personal computer
PCB	Printed circuit board
PDCP	Packet data convergence protocol
PDN	Packet data network
PDU	Protocol data unit
PHY	Physical
PoC	Proof-of-concept
QAM	Quadrature amplitude modulation
QPSK	Quadrature phase shift keying
RAN	Radio access network
RAT	Radio access technology
RF	Radio frequency
RRC	Radio resource control
RRH	Remote radio head
RRM	Radio resource management
RSSI	Received signal strength indicator
SC	Single carrier
SDU	Service data unit
STA	Station
TDD	Time division duplex
TWAG	Trusted wireless access gateway
UE	User equipment
UP	User plane
WiGig	Wireless Gigabit
WLAN	Wireless local area network

## References

[1] Association of Radio Industries and Businesses (ARIB) 2020 and Beyond Ad Hoc Group (2014). Mobile Communications Systems for 2020 and beyond. ARIB 2020 and Beyond Ad Hoc Group White Paper.

[2] (2015). G-Infrastructure PPP Vision Document, 5G Vision Brochure. http://5g-ppp.eu/wp-content/uploads/2015/02/5G-Vision-Brochure-v1.pdf.

[3] International Telecommunication Union Radiocommunication Sector (ITU-R) (2015). IMT-Vision–Framework and Overall Objectives of the Future Development of IMT for 2020 and Beyond.

[4] Flore D., Bertenyi B. (2015). “5G” Timeline in 3GPP.

[5] ABI Research ABI Research Projects 5G Worldwide Service Revenue to Reach $247 Billion in 2025. https://www.abiresearch.com/press/abi-research-projects-5g-worldwide-service-revenue/.

[6] Bryzek J. Trillion Sensors Movement in Support of Abundance and Internet of Everything. Proceedings of the VP Development, MEMS and Sensing Solutions & Chair, TSensors Summit.

[7] International Telecommunications Union Radiocommunication Sector (ITU-R) (2011). Assessment of the Global Mobile Broadband Deployments and Forecasts for International Mobile Telecommunications.

[8] International Telecommunications Union Radiocommunication Sector (ITU-R) (2015). IMT Traffic Estimates for the Years 2020 to 2030.

[9] Andrews J.G., Buzzi S., Choi W., Hanly S.V., Lozano A., Soong A.C.K., Zhang J.C. (2014). What Will 5G Be?. IEEE J. Sel. Areas Commun..

[10] International Telecommunications Union Radiocommunication Sector (ITU-R) (2014). Future Technology Trends of Terrestrial IMT Systems.

[11] MediaTek Inc. (2015). Study on Downlink Multiuser Superposition Transmission.

[12] Rangan S., Rappaport T.S., Erkip E. (2014). Millimeter-Wave Cellular Wireless Networks: Potentials and Challenges. IEEE Proc..

[13] Bhushan N., Li J., Malladi D., Gilmore R., Brenner D., Damnjanovic A., Sukhavasi R.T., Patel C., Geirhofer R. (2014). Network Densification: The Dominant Theme for Wireless Evolution into 5G. IEEE Commun. Mag..

[14] Sakaguchi K., Tran G.K., Shimodaira H., Nanba S., Sakurai T., Takinami K., Siaud I., Strinati E.C., Capone A., Karls I. (2015). Millimeter-Wave Evolution for 5G Cellular Networks. IEICE Trans. Commun..

[15] Weiler R.J., Peter M., Keusgen W., Strinati E.C., Domenico A.D., Filippini I., Capone A., Siaud I., Ulmer-Moll A., Maltsev A. Enabling 5G Backhaul and access with millimeter-waves. Proceedings of the 2014 European Conference on Networks and Communications (EuCNC).

[16] International Telecommunication Union Radiocommunication Sector (ITU-R) (2015). Technical Feasibility of IMT in Bands above 6 GHz.

[17] NGMN Alliance Backhaul Provisioning for LTE-Advanced & Small Cells. https://www.ngmn.org/uploads/media/150929_NGMN_P-SmallCells_Backhaul_for_LTE-Advanced_and_Small_Cells.pdf.

[18] Hur S., Kim T., Love D.J., Krogmeier J.V., Thomas A. (2013). Millimeter Wave Beamforming for Wireless Backhaul and Access in Small Cell Networks. IEEE Trans. Commun..

[19] Pi Z., Choi J., Heath R. (2016). Millimeter-Wave Gigabit Broadband Evolution Toward 5G: Fixed Access and Backhaul. IEEE Commun. Mag..

[20] Visentin T., Keusgen W., Weiler R.J. Dual-Polarized Square-Shaped Offset-Fed Reflectarray Antenna with High Gain and High Bandwidth in the 60 GHz Domain. Proceedings of the 2015 9th European Conference on Antennas and Propagation (EUCAP).

[21] Maltsev A., Sadri A., Pudeyev A., Bolotin I. (2016). Highly Directional Steerable antennas. IEEE Veh. Technol. Mag..

[22] MiWEBA Website. http://www.miweba.eu/.

[23] Ishii H., Kishiyama Y., Takahashi H. A novel architecture for LTE-B: C-plane/U-plane split and phantom cell concept. Proceedings of the IEEE Globecom Workshops.

[24] Inoue M., Wu G., Hase Y., Sugitani A., Kawakami E., Shimizu S., Tokuda K. (2000). An IP-Over-Ethernet-Based Ultrahigh-Speed Wireless LAN Prototype Operating in the 60-GHz Band. IEICE Trans. Commun..

[25] IEEE Standards Association (2009). IEEE Standard for Information Technology—Telecommunications and Information Exchange between Systems—Local and Metropolitan Area Networks—Specific Requirements—Part 15.3: Amendment 2: Millimeter-Wave-Based Alternative Physical Layer Extension.

[26] Okada K., Kondou K., Miyahara M., Shinagawa M., Asada H., Minami R., Yamaguchi T., Musa A., Tsukui Y., Asakura Y. (2013). Full Four-Channel 6.3-Gb/s 60-GHz CMOS Transceiver With Low-Power Analog and Digital Baseband Circuitry. IEEE J. Solid-State Circuits.

[27] Saito N., Tsukizawa T., Shirakata N., Morita T., Tanaka K., Sato J., Morishita Y., Kanemaru M., Kitamura R., Shima T. (2013). A Fully Integrated 60-GHz CMOS Transceiver Chipset Based on WiGig/IEEE802.11ad With Built-in Self Calibration for Mobile Usage. IEEE J. Solid-State Circuits.

[28] Sakaguchi K., Mohamed E.M., Kusano H., Mizukami M., Miyamoto S., Rezagah R.E., Takinami K., Takahashi K., Shirakata N., Peng H. (2015). Millimeter-Wave Wireless LAN and Its Extension toward 5G Heterogeneous networks. IEICE Trans. Commun..

[29] IEEE Standards Association (2012). IEEE Standard for Information Technology—Telecommunications and Information Exchange between Systems—Local and Metropolitan Area Networks—Specific Requirements Part 11: Wireless LAN Medium Access Control (MAC) and Physical Layer (PHY) Specifications Amendment 3: Enhancements for Very High Throughput in the 60 GHz Band.

[30] Wireless Gigabit (WiGig) Alliance (2010). WiGig MAC and PHY Specification.

[31] Intel Corporation Intel^®^ Wireless Docking Overview. http://www.intel.com/content/www/us/en/wireless-products/wireless-docking.html.

[32] Snapdragon Staff Get to Know the Snapdragon 810 Processor. https://www.qualcomm.com/news/snapdragon/2014/12/02/get-know-snapdragon-810-processor.

[33] TP-LINK Technologies Co., Ltd. Talon AD7200 Multi-Band Wi-Fi Router. http://www.tp-link.com/en/products/details/AD7200.html.

[34] Samsung Electronics Co., Ltd. Samsung Electronics and Deutsche Telekom Demonstrate World’s First End-to-End 5G Solution at Mobile World Congress 2016. https://news.samsung.com/global/samsung-electronics-and-deutsche-telekom-demonstrate-worlds-first-end-to-end-5g-solution-at-mobile-world-congress-2016.

[35] Branda M. Qualcomm Research Demonstrates Robust mmWave Design for 5G. https://www.qualcomm.com/news/onq/2015/11/19/qualcomm-research-demonstrates-robust-mmwave-design-5g.

[36] Samsung Electronics Co., Ltd. 5G Vision White Paper. http://www.samsung.com/global/business-images/insights/2015/Samsung-5G-Vision-0.pdf.

[37] Huawei Technologies Co., Ltd. Huawei to Bring 73 GHz mmWave Mu-MIMO live Demo to Deutsche Telekom. http://www.huawei.com/en/news/2016/2/73GHzmm-Wave-Mu-MIM-live-demo.

[38] Nokia Networks Nokia Networks Showcases 5G speed of 10Gbps with NI at the Brooklyn 5G Summit. http://networks.nokia.com/news-events/press-room/press-releases/nokia-networks-showcases-5g-speed-of-10gbps-with-ni-at-the-brooklyn-5g-summit.

[39] Goovaerts D. Verizon, T-Mobile Seek Permission to Test 5G Tech at 28 GHz. http://www.wirelessweek.com/news/2016/03/verizon-t-mobile-seek-permission-test-5g-tech-28-ghz.

[40] InterDigital InterDigital, Imec and Peraso Demonstrate World’s First WiGig-Based Millimeter Wave Mesh Backhaul System. http://ir.interdigital.com/releasedetail.cfm?releaseid=827608.

[41] NEC Corporation NEC launches New Millimeter Wave Radio for 5G Backhauling. http://www.nec.com/en/press/201602/global_20160210_01.html.

[42] 3rd Generation Partnership Project (3GPP) (2014). Small Cell Enhancements for E-UTRA and E-UTRAN; Higher Layer Aspects.

[43] Nokia Networks White Paper, LTE-Advanced Carrier Aggregation Optimization. http://networks.nokia.com/sites/default/files/document/nokia_carrier_aggregation_white_paper.pdf.

[44] Lauridsen M., Wang H., Mogensen P. LTE UE Energy Saving by Applying Carrier Aggregation in a HetNet scenario. Proceedings of the 2013 IEEE 77th Vehicular Technology Conference (VTC Spring).

[45] Mohamed E.M., Sakaguchi K., Sampei S. Delayed offloading using cloud cooperated millimeter wave gates. Proceedings of the 2014 IEEE 25th Annual International Symposium on Personal, Indoor, and Mobile Radio Communication (PIMRC).

[46] 4G Americas White Paper, LTE Aggregation & Unlicensed Spectrum, 2015. http://www.4gamericas.org/.

[47] Burbidge R. (2016). Liaison from 3GPP on LWA and LWP.

[48] Francois R. ITU-R Administrative Circular CA/226. Proceedings of the First Session of the Conference Preparatory Meeting for WRC-19 (CPM19-1).

[49] 3rd Generation Partnership Project (3GPP) (2016). Evolved Universal Terrestrial Radio Access (E-UTRA); User Equipment (UE) Radio Transmission and Reception (Release 13).

[50] Yong S.K., Xia P., Garcia A.V. (2011). 60 GHz Technology for Gbps WLAN and WPAN.

[51] Cordeiro C. (2015). 802.11 NG60 SG Proposed PAR.

[52] Intel Product Brief, Intel^®^ Tri-Band Wireless-AC 17265. http://www.intel.com/.

[53] 3rd Generation Partnership Project (3GPP) (2016). Architecture Enhancement for Non-3GPP Access.

[54] IEEE Standards Association (2009). IEEE Standard for Information Technology—Telecommunications and Information Exchange between Systems–Local and Metropolitan Area Networks—Specific Requirements—Part 3: CSMA/CD Access Method and Physical Layer Specifications Amendment 3: Data Terminal Equipment (DTE) Power via the Media Dependent Interface (MDI) Enhancements.

[55] 3rd Generation Partnership Project (3GPP) (2016). Access to the 3GPP Evolved Packet Core (EPC) via Non-3GPP Access Networks.

[56] 3rd Generation Partnership Project (3GPP) (2016). Access Network Discovery and Selection Function (ANDSF) management object (MO).

[57] Cai Y., Thomas M., Li M., Xia J., Giannelli C. (2010). Mobile Wireless Middleware, Operating Systems, and Applications.

[58] Hu Y.C., Patel M., Sabella D., Sprecher N., Young V. (2015). Mobile Edge Computing—A Key Technology towards 5G.

[59] Peng H., Yamamoto T., Nanba S. (2015). LTE/WiGig RAN-level interworking architecture for 5G millimeter-wave heterogeneous networks. IEICE Trans. Commun..

[60] Huang J., Encinar J.A. (2007). Reflectarray Antennas.

[61] Sun S., Rappaport S., Heath R.W., Andrew N., Rangan S. (2014). MIMO for Millimeter-wave Wireless Communications: Beamforming, Spatial Multiplexing, or Both?. IEEE Commun. Mag..

[62] Maltsev A., Sadri A., Pudeyev A., Bolotin I., Davydov A., Morozov G., Weiler R.J. Partially Adaptive Arrays application for MU-MIMO mode in a MmWave Small Cells. Proceedings of the 2015 IEEE 26th Annual International Symposium on Personal, Indoor, and Mobile Radio Communications (PIMRC).

[63] Fernandes C.A. (1999). Shaped dielectric lenses for wireless millimeter-wave communications. IEEE Antennas Propag. Mag..

[64] Karttunen A., Ala-Laurinaho J., Sauleau R., Räisänen A.V. A Study of Extended Hemispherical Lenses for a High-Gain Beam-Steering Antenna. Proceedings of the 4th IEEE European Conference on Antennas and Propagation.

[65] Artemenko A., Maltsev A., Mozharovskiy A., Maselennikov R., Sevastyanov A., Ssorin V. (2013). Millimeter-wave electronically steerable integrated lens antennas for WLAN/WPAN applications. IEEE Trans. Antennas Propag..

[66] European Telecommunications Standards Institute (ETSI) (2014). Open Radio Equipment Interface (ORI): Requirements for Open Radio Equipment Interface (ORI).

[67] European Telecommunications Standards Institute (ETSI) (2014). Open Radio Equipment Interface (ORI): Interface Specification. Par 1: Low Layers (Release 4).

[68] Open vSwitch Website. http://openvswitch.org/.

[69] Iperf. https://iperf.fr/.

[70] MiWEBA Proof of Concept. https://www.youtube.com/watch?v=qTPv13c7n34.

[71] Tran G.K., Shimodaira H., Rezagah R., Sakaguchi K., Araki K. Dynamic cell activation and user association for green 5G heterogeneous cellular networks. Proceedings of the 2015 IEEE 26th Annual International Symposium on Personal, Indoor, and Mobile Radio Communications (PIMRC).

[72] Tran G.K., Shimodaira H., Rezagah R., Sakaguchi K., Araki K. Practical evaluation of on-demand smallcell on/off based on traffic model for 5G cellular networks. Proceedings of the IEEE WCNC 2016.

